# Liver as a Source for Thymidine Phosphorylase Replacement in Mitochondrial Neurogastrointestinal Encephalomyopathy

**DOI:** 10.1371/journal.pone.0096692

**Published:** 2014-05-06

**Authors:** Elisa Boschetti, Roberto D’Alessandro, Francesca Bianco, Valerio Carelli, Giovanna Cenacchi, Antonio D. Pinna, Massimo Del Gaudio, Rita Rinaldi, Vincenzo Stanghellini, Loris Pironi, Kerry Rhoden, Vitaliano Tugnoli, Carlo Casali, Roberto De Giorgio

**Affiliations:** 1 Department of Surgical and Medical Sciences, University of Bologna, Bologna, Italy; 2 Department of Biomedical and Neuromotor Sciences, University of Bologna, Bologna, Italy; 3 Institute of Neurological Sciences, University of Bologna, Bologna, Italy; 4 Neurology Unit, St. Orsola-Malpighi Hospital, Bologna, Italy; 5 Department of Medico-Surgical Sciences and Biotechnologies, University ‘La Sapienza’, Rome, Italy; Auburn University, United States of America

## Abstract

Mitochondrial neurogastrointestinal encephalomyopathy (MNGIE) is a rare autosomal recessive mitochondrial disease associated with mutations in the nuclear *TYMP* gene. As a result, the thymidine phosphorylase (TP) enzyme activity is markedly reduced leading to toxic accumulation of thymidine and therefore altered mitochondrial DNA. MNGIE is characterized by severe gastrointestinal dysmotility, neurological impairment, reduced life expectancy and poor quality of life. There are limited therapeutic options for MNGIE. In the attempt to restore TP activity, allogenic hematopoietic stem cell transplantation has been used as cellular source of TP. The results of this approach on ∼20 MNGIE patients showed gastrointestinal and neurological improvement, although the 5-year mortality rate is about 70%. In this study we tested whether the liver may serve as an alternative source of TP. We investigated 11 patients (7M; 35–55 years) who underwent hepatic resection for focal disorders. Margins of normal liver tissue were processed to identify, quantify and localize the TP protein by Western Blot, ELISA, and immunohistochemistry, and to evaluate *TYMP* mRNA expression by qPCR. Western Blot identified TP in liver with a TP/GAPDH ratio of 0.9±0.5. ELISA estimated TP content as 0.5±0.07 ng/μg of total protein. TP was identified in both nuclei and cytoplasm of hepatocytes and sinusoidal lining cells. Finally, *TYMP* mRNA was expressed in the liver. Overall, our study demonstrates that the liver is an important source of TP. Orthotopic liver transplantation may be considered as a therapeutic alternative for MNGIE patients.

## Introduction

Mitochondrial neurogastrointestinal encephalomyopathy (MNGIE) is a rare autosomal recessive mitochondrial disease due to mutations in the nuclear *TYMP* gene encoding thymidine phosphorylase (TP). This enzyme converts the nucleosides thymidine (dThd) and deoxyuridine into thymine and uracil, respectively [1]. *TYMP* mutations lead to a marked reduction (or virtual absence) of TP activity [2] resulting in a toxic accumulation of nucleosides in plasma of MNGIE patients. This biochemical imbalance leads to secondary mitochondrial DNA (mtDNA) point mutations, multiple deletions and, more importantly, mtDNA depletion [3], [4]. The nucleotide pool for mtDNA replication depends on the salvage pathway. In MNGIE, excess dThd enters mitochondria and competes with deoxycytidine for thymidine kinase 2, becoming its predominant substrate and causing mtDNA abnormalities [5], [6]. In tissues with high cell turnover and active proliferation, such as bone marrow or liver, dThd accumulation is prevented with a rapid equilibration of nucleotides between the cytosol and the mitochondrial matrix. On the opposite, post-mitotic, high-energy dependent tissues, such as brain, skeletal and smooth muscle, rely only on the nucleotide salvage pathway and are therefore the target for MNGIE [5], [6], [7], [8]. Since nuclear and mitochondrial nucleotide pools originate from different pathways, *TYMP* mutations do not influence nuclear DNA turnover [5].

From a clinical standpoint, MNGIE is characterized by severe gastrointestinal symptoms and frequent intestinal sub-occlusive episodes (i.e. chronic intestinal pseudo-obstruction) [9] due to marked impairment of gut motility; in addition, other common features include ptosis and ophthalmoparesis, cachexia, peripheral neuropathy, myopathy, leukoencephalopathy (detectable by MRI), and lactic acidosis [10],[11]. Clinical manifestations differ depending on the degree of the TP defect. Indeed, typical MNGIE patients have ∼5% residual TP activity, experience major symptoms from the second decade, and their overall life expectancy is limited to the fourth decade. In contrast, patients with a partial loss of TP function (∼10–15% residual TP activity) manifest symptoms later at an adult age and their life expectancy is beyond the fifth decade. Notably, MNGIE relatives carrying heterozygous *TYMP* mutations never manifest the syndrome. Since TP is a homodimer, the presence of a mutant allele leads to the formation of only 25% wild-type TP molecules, while 75% of dimers contain at least one dysfunctional monomer. Asymptomatic heterozygous subjects have ∼25–35% residual TP activity and this threshold may represent the target for therapeutic purposes [12], [13].

So far, there are no established therapeutic options for patients with MNGIE. Peritoneal dialysis is a commonly used approach to lower plasma concentrations of toxic nucleosides in order to reduce the clinical manifestations of the disease, and in particular the gastrointestinal symptoms (e.g. vomiting, abdominal pain, and weight loss). This approach, however, produces only a transient benefit since clearance lasts for only a few hours [14], [15]. Another strategy is based on the replacement of TP activity using cells containing adequate levels of the enzyme. Platelet infusion has been used to this end although with limited success [16], and erythrocyte encapsulated TP is under clinical development [17]. Gene therapy may be also a valuable option. TP-deficient B-lymphoblastoid cells from MNGIE patients and partially myeloablated double *TYMP/UPP1* knockout mice have been transfected with lentiviral or adeno-associated virus (AAV) vectors carrying the *TYMP* coding sequence with a reduction in nucleoside concentrations [18], [19]. The effectiveness of *TYMP* gene therapy, however, still awaits confirmation in *ad hoc* clinical trials. Finally, allogenic hematopoietic stem cell transplantation (AHSCT) has been performed to provide a permanent cellular source of TP in MNGIE patients [20]. So far, the worldwide experience has shown some positive results characterized by symptomatic improvement, increased TP activity and reduced dThd and deoxyuridine blood levels [21]. However, AHSCT is dramatically limited by an overall mortality, that has been demonstrated to be ∼70% [22], (Hirano et al., Child Neurology Society's annual meeting, 2013, Austin, TX).Based on this experience, organ transplantation, other than AHSCT, may represent an alternative option for treating patients with MNGIE. Since the liver is the main organ for protein biosynthesis and the transplantation success is estimated at ∼90% of cases [23], the present study has been designed to test whether the liver can be proposed as a source of TP. Herein we provide evidence that liver is a good source of TP and suggest that MNGIE patients might benefit from orthotopic liver transplantation.

## Materials and Methods

### Tissue Sampling

Hepatic tissue samples (1×1 cm), were obtained from eleven subjects (7M, 32–67 years), undergoing open surgery for neoplastic (primary hepato-cellular carcinoma) liver disease. Tissue samples were harvested in a macroscopically normal area and the histopathological analysis confirmed a normal liver histology. Collected specimens were processed as follows: a) five samples were immediately frozen in RNA*later* RNA Stabilization Reagent (Sigma Aldrich, Milan, Italy) and stored at −80°C; b) six samples were formalin fixed and paraffin embedded.

### Ethical Considerations

The Institutional Review Board of the St. Orsola-Malpighi University Hospital Ethics Committee approved this research project (31/2013/U/OssN Prot.nr.1380/2013), which complied with the Declaration of Helsinki. A written informed consent was obtained from each participant and anonymized samples were collected as ‘MNGIE-(consecutive number)-age-sex’ or ‘NON-MNGIE-(consecutive number)-age-sex’.

### Protein extraction

Total protein was extracted from five liver tissue samples (0.5 g) (3 M) using tissue protein extraction reagent in the presence of a protease inhibitor cocktail (Thermo Scientific, Milan, Italy) according to the manufacturer's instructions. Protein fractions were quantified for total protein content using a NanoDrop 2000 spectrophotometer (Thermo Scientific, Milan, Italy), and were stored at −80°C. Total protein extracts from our laboratory archive (obtained with the same technique) served as controls. Specifically, total protein was extracted from bone marrow (n = 3, 2 M; 40–65 years), duodenal mucosa (n = 3, 1 M; 38–57 years), skeletal muscles (n =  3, 2 M; 33–61 years), and non-MNGIE (n =  3, 1 M; 33–61 years) and MNGIE buffy coats (n = 1, female aged 29 years).

### Western blot (WB) analysis

Protein separation was carried out on 50 µg samples of total protein in a 12% tris-glycine gel (Thermo Scientific, Milan, Italy). Protein was transferred onto nitrocellulose membrane (Macherrey-Nagel, Düren, Germany) overnight at 12 mV. Membranes were blocked with a buffer containing 5% fat-free milk and then incubated overnight at 4°C with a mouse anti-TP primary antibody (Thermo Scientific, Milan, Italy) at a final concentration of 2 µg/ml. Membranes were washed three times and incubated with an anti-mouse peroxidase-conjugated secondary antibody (Sigma Aldrich, Milan, Italy). Immunoreactive bands were visualized by enhanced chemiluminescence (GE Healthcare, Buckinghamshire, UK) on a ChemiDoc MP System and quantified by Image Lab software version 4.0 (Bio-Rad Laboratories, Hercules, CA, USA). Band intensities were expressed relative to total protein and/or to the intensity of GAPDH detected on the same membrane following stripping with Restore Plus Western Blot Stripping Buffer (Thermo Fisher Scientific, Pittsburgh, PA, USA) and overnight incubation with GAPDH antibody at 4°C at a 1∶1000 dilution (Abcam, Cambridge, UK). Each assay was conducted in technical triplicate.

### ELISA TP quantification

TP amount was measured on total protein extracts from the same 5 subjects included in the WB separation, using an ELISA Kit for human TP (Uscn Life Science Inc., Wuhan, China) according to the manufacturer's instructions. A quantity of 10 µl of protein extract (5 µg/μl of total protein) for each sample was assayed in triplicate. When reactions were complete, multi-well ELISA pre-coated plates were read in an Infinite M200 multi plate reader (TECAN, Männedorf, Switzerland) at λ 450 nm. TP concentrations in each sample were estimated from a TP standard curve. Assays were performed in duplicate.

### Immunohistochemical analysis

Anti-TP immunohistochemical analysis was performed on formalin-fixed, paraffin-embedded liver samples from 6 subjects. Sections of normal liver tissue, obtained from the laboratory histology archive, were used as controls. Additionally, duodenum (n = 3) and skeletal muscles (n = 3) (from 6 different subjects, 4 M; 32–67 years) were processed as positive and negative controls, respectively. Moreover, paraffin-fixed liver, duodenum, and skeletal muscle tissue samples from one MNGIE patient (male; 37 years) were included.

Tissue sections were deparaffinized in xylene and rehydrated through graded ethanol (Carlo Erba, Milan, Italy). Antigen retrieval was carried out by heating sections in a 90°C water for 25 min in the presence of 10 mmol/l sodium citrate buffer pH 6.0 (Carlo Erba, Milan, Italy). Sections were treated with an endogenous peroxidase blocking kit (Gene Tex, Aachen, Germany). Subsequent steps were performed using a commercial kit (Millipore, Milan, Italy) following the manufacturer's instructions. Sections were incubated with mouse primary anti-TP antibody at a final concentration of 0.002 µg/μl (Abcam, Cambridge, UK), in a humidified chamber overnight at 4°C. After dehydration slides were cover-slipped using DPX (Sigma-Aldrich, Milan, Italy).

### RNA Extraction

Liver tissue samples (30 mg) (n = 6, 3M, aged 32–67 years) were mechanically disrupted with sterile scissors and homogenized using QIAshredder according to the manufacturer's instructions. RNA was extracted using RNeasy mini kit (Qiagen, Hilden, Germany) and eluted in a final volume of 60 µl. Sample purity was assessed with a NanoDrop 2000 spectrophotometer (Thermo Scientific, Milan, Italy). Total RNA extracts from healthy donors, used as controls, were purchased from BioChain (Milan, Italy) and included: bone marrow (n = 6, 3 M, aged 27–65 years), duodenal mucosa (n = 6, 4 M aged 31–63 years), and skeletal muscle (n = 6, 3 M aged 24–65 years).

### Reverse Transcription (RT)

RT was performed on 500 ng of total RNA in a 20 µl total reaction volume using Quantitect reverse transcription kit (Qiagen, Hilden, Germany). Samples were incubated for 2 min at 42°C with gDNA Wipeout Buffer to avoid possible genomic DNA contamination. RT conditions were: 15 min 45°C, 3 min 95°C and 5 min 4°C. Each sample was reverse transcribed twice and the obtained cDNA stored at −20°C.

### qPCR assay

Relative gene expression analysis was performed on an Applied Biosystem 7500 Fast real time PCR system (Life Technologies, Milan, Italy) by two step qPCR assays using Quanti Fast Probe Assay Duplex Detection (Qiagen, Hilden, Germany) and following MIQE guidelines [24]. Amplification was performed in a 25 µl final volume including 2 µl of cDNA as template. Each sample was assayed in triplicate and the analysis was duplicated using cDNA from two independent RT reactions. The PCR Master Mix was prepared according to the manufacturer's instructions in the presence of High-ROX dye Solution. Amplification conditions were: 5 min at 95°C followed by 40 cycles (95°C for 30 sec, 60°C for 30 sec). Amplicon length was assessed using 2% agarose gel electrophoresis using SYBR green 1X (Invitrogen, Paisley, UK). Primer probes (Qiagen, Hilden, Germany), optimized for use with the Quanti Fast Probe Assay Duplex Detection Kit, were Hs_TYMP_1_FAM (QF00274225) for TYMP and Hs_ACTB_2_MAX (QF00531209) for *actin-β* Data were reported as ΔΔC_T_ using *actin-β* as a reference since the mRNA transcript for this gene was similar among the selected human tissues. Bone marrow was the calibrator tissue at unit value.

### Statistical analysis

Differences between samples analyzed by WB, ELISA and qPCR, were detected by the One Way ANOVA non-parametric test followed by Tukey's post-test.

## Results

### TP analysis and quantification

**Figure 1:**
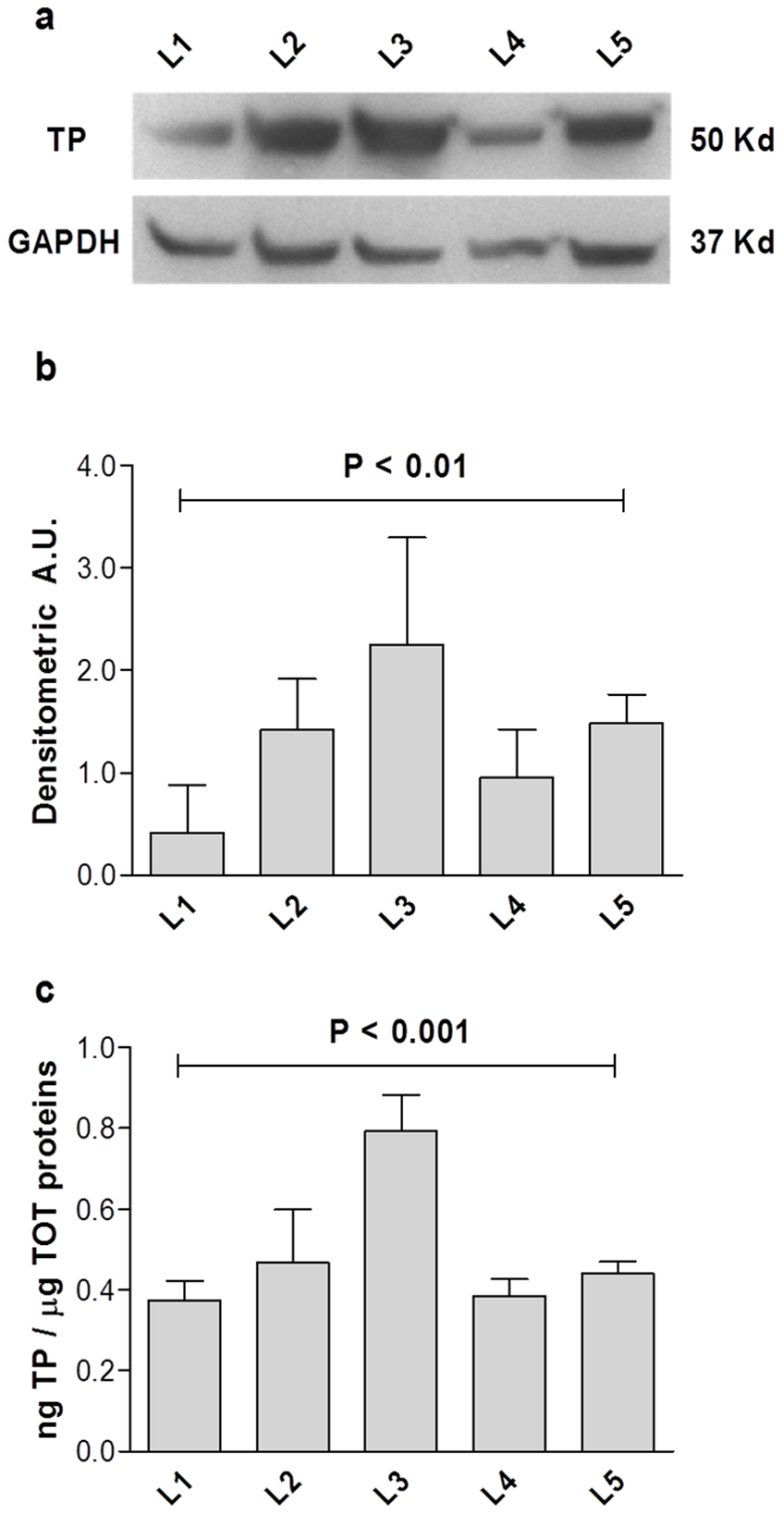
TP occurrence and concentration in control liver. Figure 1A shows an example of a WB separation of 50 ng total protein from healthy liver. TP and reference protein GAPDH chemioluminescence are reported from liver L1 to L5. Figure 1B illustrates the densitometric arbitrary units (A.U.) calculated normalizing TP chemioluminescent signal on the internal reference protein GAPDH ± SD and showing a significant variability among non-MNGIE subjects (P< 0.01, one way non parametric test ANOVA). The graph in Figure 1C reports the TP concentration measured by ELISA and expressed as ng TP/μg total proteins for the 5 liver tissue samples ± SD and showing a significant variability among non-MNGIE subjects (P< 0.001, one way non parametric test ANOVA).

The presence and the amount of TP in healthy human liver tissues were assessed by WB and ELISA (Figure 1). To avoid artifacts assays were performed using independent protein extractions as starting material from five subjects and the results obtained with the two techniques were concordant. Figure 1A shows that TP protein is expressed in all samples. The densitometric arbitrary units (AU), obtained normalizing TP chemioluminescent signal on the internal reference protein GAPDH, is illustrated in Figure 1B. The mean densitometric ratio TP/GAPDH is 0.9±0.5 AU and TP expression varies significantly among subjects (P<0.01). TP quantification, expressed as ng TP/μg total protein, is obtained with ELISA (Figure 1C). The average of TP content is 0.5 ng/μg total protein, ranging from 0.4 to 0.75 ng/μg total protein. The variation in TP expression among subjects is confirmed by ELISA (P<0.001).

### TP localization in liver and in a selected human tissue panel

**Figure 2:**
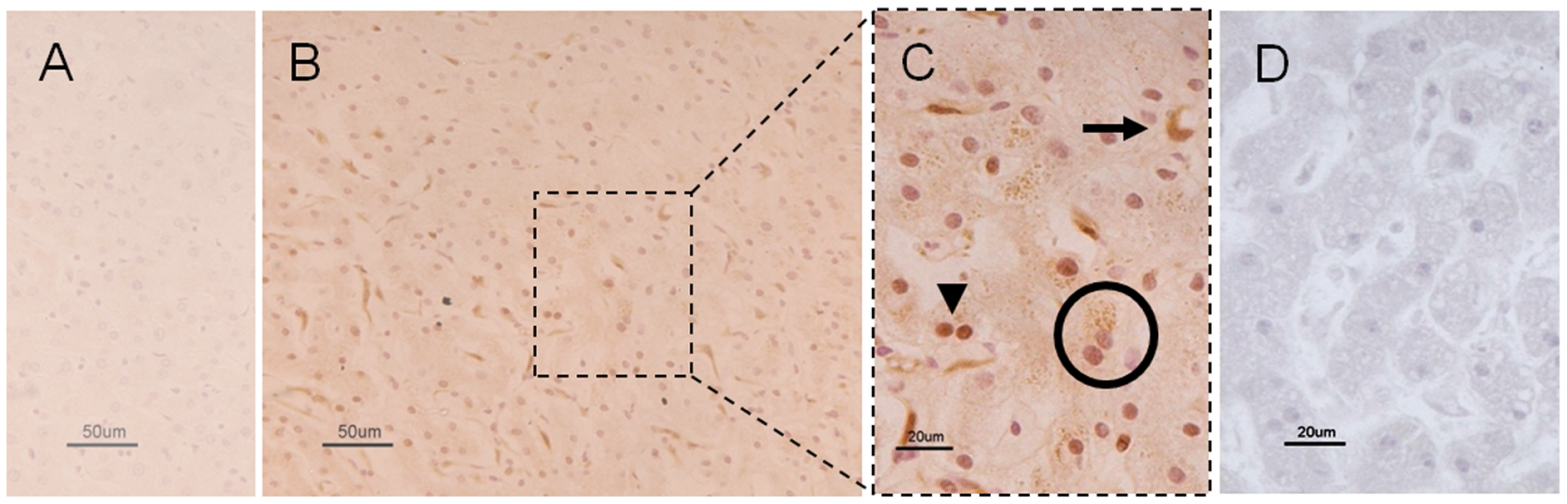
Representative photomicrographs showing TP immunoreactivity in control and MNGIE liver. Figure 2A demonstrates the lack of TP immunolabeling in a normal liver section in which the primary antibody was omitted (negative control). Figure 2B (low magnification) and 2C (high magnification) illustrate TP immunoreactivity in a normal liver section. In Figure 2C, the arrowhead and circle, (Figure 2C) point to TP immunostained nuclei and cytoplasm of hepatocytes, while the arrow indicates non-hepatocytic cells with features of bile duct elements. Also, note the lack of any TP immunolabeling in a MNGIE liver section (Figure 2D). Calibration bars  = 20 µm and 50 µm in 2C, 2D and 2A, 2B, respectively.

Figure 2 demonstrates the TP immunoreactivity in liver tissues of controls (A-C) and in a MNGIE patient (D). Compared to MNGIE liver tissue, which lacked TP immunostaining (Figure 2D), TP immunoreactivity was clearly detected in the cytoplasm as well as nuclei of hepatocytes in control tissues (Figures 2B-C). In addition, some sinusoidal lining cell resembling Kupffer cells showed TP immunolabeling (Figures 2B-C).

**Figure 3:**
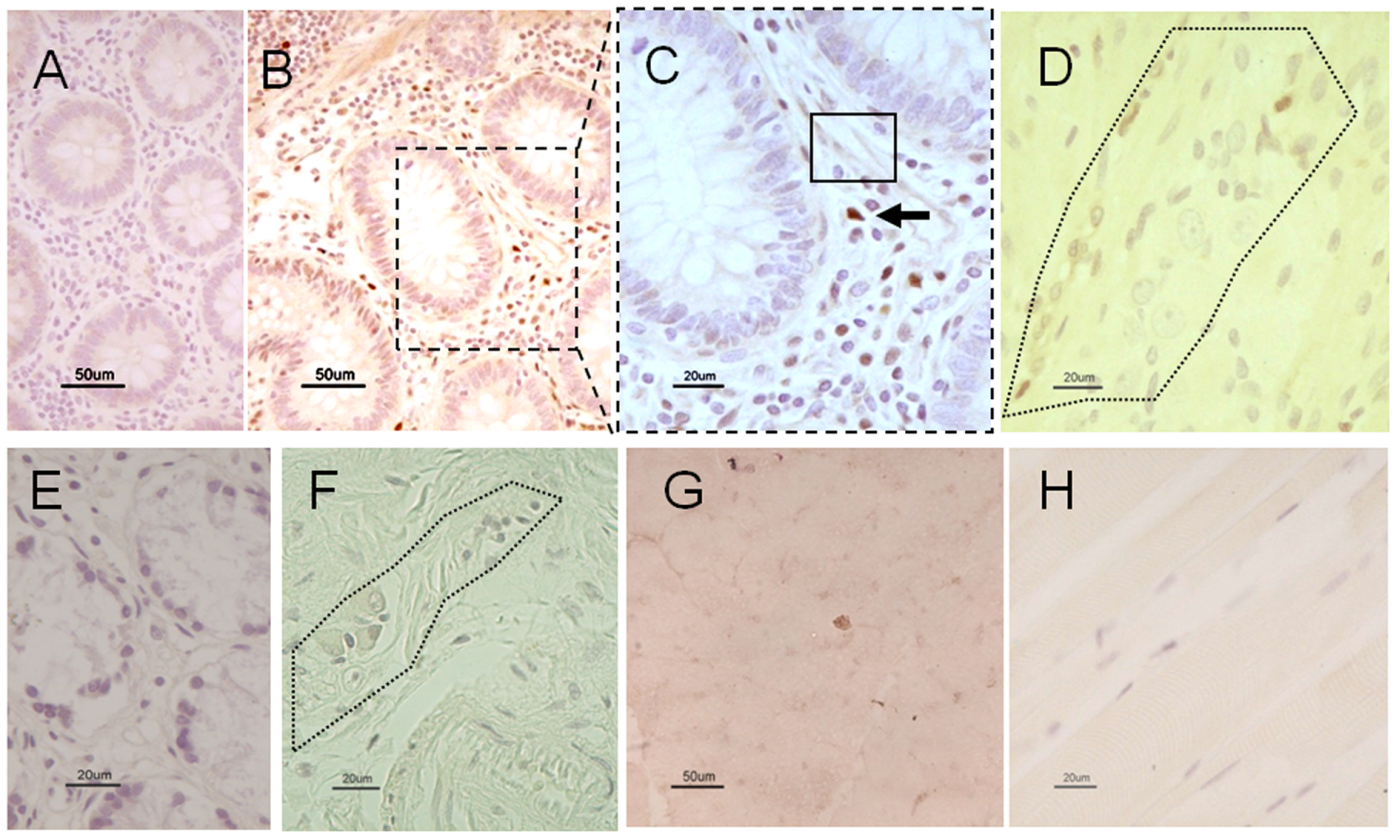
TP immunoreactivity in the duodenum and skeletal muscle of control and in a MNGIE patient. TP immunolabeling is lacking in a tissue section of normal duodenal mucosa in which primary antibody was omitted (negative control) (Figure 3A). Figure 3B (low magnification) and 3C (high magnification) show TP immunolabeling in the control mucosa. The black arrow indicates TP immunolabeled cells with features of immunocytes distributed throughout the *lamina propria*; the rectangle shows a less intense TP immunostaining in the cytoplasm of cells with features of fibroblasts. The dotted line area in figure 3D indicates a myenteric plexus displaying TP immunolabelling in non-neuronal cells (likely glial cells). Note the lack of TP immunolabeling in the mucosal *lamina propria* (Figure 3E) and myenteric plexus (dotted line) of a MNGIE patient (Figure 3F). The TP immunolabeling was negative also in normal (Figure 3G) and MNGIE (Figure 3H) skeletal muscle. Calibration bars  = 20 µm and 50 µm in 3C, 3D, 3E, 3F, 3H and 3A, 3B, 3G respectively.

TP localization was also identified in a panel of selected human tissues (Figure 3). In the duodenal mucosa, TP immunoreactivity was revealed in the cytoplasm of cells reminiscent of immunocytes normally distributed throughout the *lamina propria* (Figures 3 A-C). Also, TP immunolabeling was detected in the duodenal neuromuscular compartment, specifically in non-neuronal cells (likely glial cells) of the myenteric plexus (Figure 3 D). As expected, TP immunolabeling was not identified in any cell of the duodenal mucosal *lamina propria* and myenteric plexus of a MNGIE patient (Figures 3 E, F). Finally, both normal (Figure 3 G) (negative control) and MNGIE skeletal muscle (Figure 3 H) lacked TP immunolabeling.

### TP abundance in a selected human tissue panel

**Figure 4:**
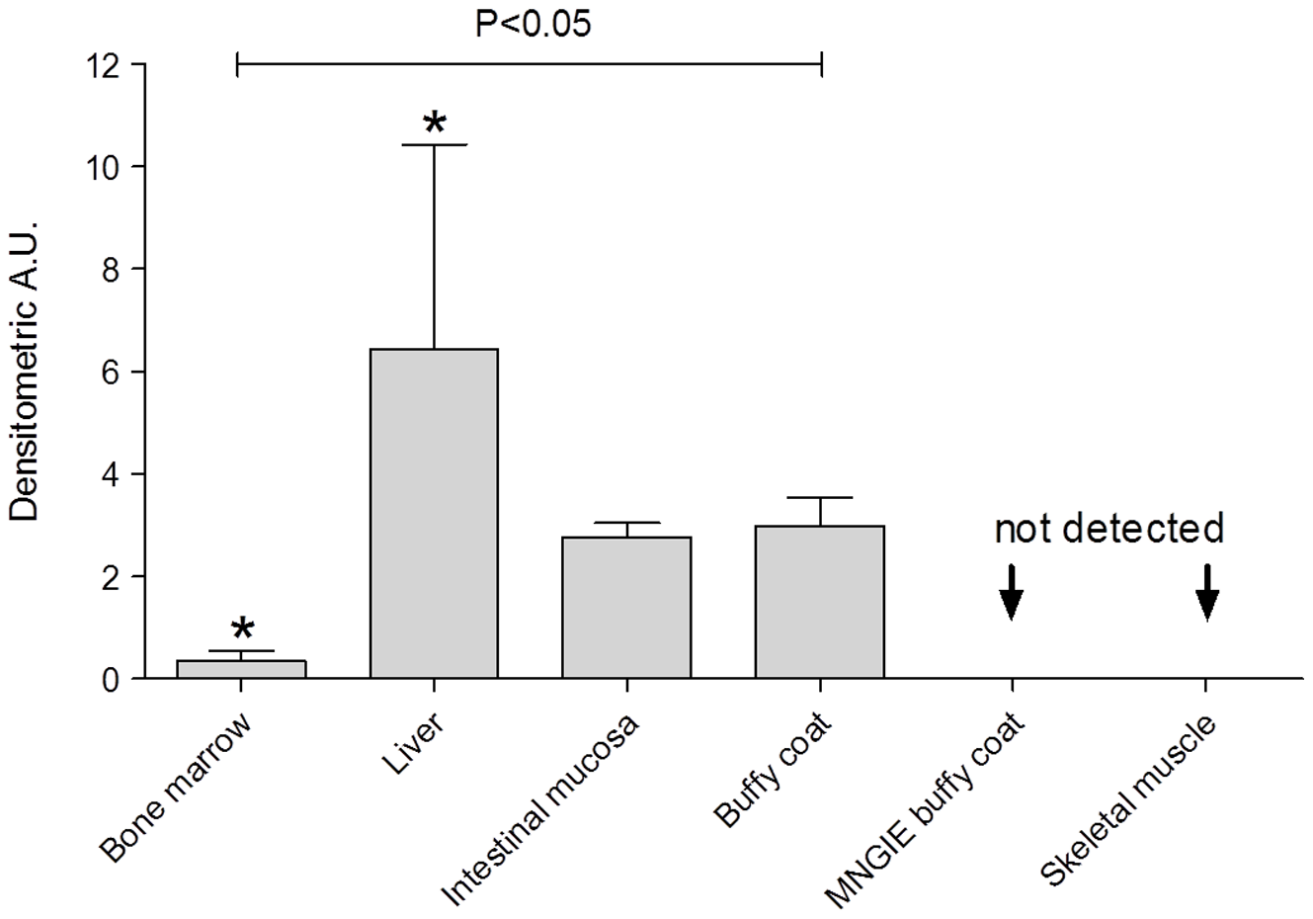
TP concentration and distribution in a panel of selected human tissues. The graph reports the A.U. calculated normalizing TP chemioluminescent signal on total proteins. Data are reported as mean ± SD using bone marrow as calibrator tissue. The TP concentrations vary significantly among different tissues (P< 0.05 one way ANOVA non-parametric test; *P< 0.05 Tukey's post-test).

Quantitative TP expression in human liver was compared to that of other tissues based on previously published animal models (Figure 4) [25]. Control liver samples have TP densitometric values six times higher than bone marrow samples (P<0.05), whereas normal buffy coats and intestinal mucosa have TP levels intermediate between liver and bone marrow (neither reached statistical significance). As expected, TP is not detectable in negative controls, i.e. normal skeletal muscle, or buffy coat of a patient with MNGIE. TP concentrations vary significantly among tissues containing the enzyme (P<0.05).

### qPCR screening

**Figure 5:**
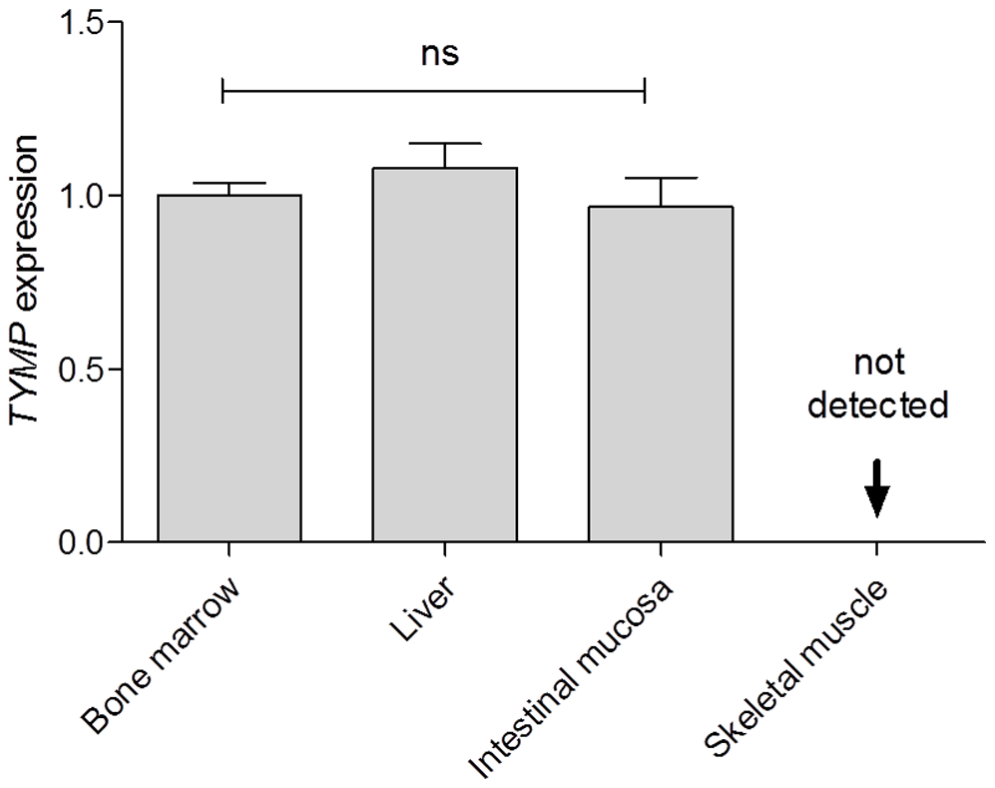
*TYMP* mRNA transcription in a panel of healthy human tissues. The amount of *TYMP* mRNA has been reported ± SD. Bone marrow was used as a calibrator tissue. *TYMP* mRNA expression did not vary significantly in bone marrow, liver, and duodenal mucosa. No *TYMP* transcript was found in skeletal muscle.

*TYMP* mRNA levels in bone marrow, liver, duodenum, and skeletal muscle were investigated by qPCR (Figure 5). Bone marrow, liver, and duodenum expressed *TYMP* at a comparable level, whereas skeletal muscle did not express any *TYMP* mRNA.

## Discussion

In this study, we assessed the expression of TP in normal human liver in comparison with a number of other control as well as MNGIE tissues. The results unequivocally demonstrate that the normal liver expresses *TYMP* and markedly synthesizes TP, suggesting that this organ is a possible option for transplantation in MNGIE patients.

So far, two main non-invasive strategies have been attempted to reduce circulating toxic levels of dThd, i.e. peritoneal dialysis [3] and platelet infusion [16]. These therapeutic approaches, however, have demonstrated only short term beneficial effects in MNGIE patients, paving the way for tissue transplantation as a permanent source of TP. Long term TP replacement is thought to reduce dThd accumulation in plasma and tissues thereby preventing the progression of MNGIE-related clinical manifestations. In support of this concept, heterozygous subjects for *TYMP* mutations have ∼30% of TP residual activity which is sufficient to avoid the MNGIE phenotype [26]. AHSCT has proven to be valuable to permanently restore TP function, reduce dThd and mitigate symptoms in MNGIE patients. So far ∼20 cases of MNGIE have undergone AHSCT, although the 5-year overall mortality is the ∼50% [22] and increasing up to ∼70% in recently presented results (Hirano et al., Child Neurology Society's annual meeting, 2013, Austin, TX).

In order to find a new permanent source of TP, possibly with a lower risk of mortality associated with transplantation, we considered the liver as a potentially useful tissue because of its physiological roles and transplantation outcome. First of all, the liver is the prototype organ for protein biosynthesis, trafficking and release; secondly, orthotopic liver transplantation has been found to be successful in 90% of cases [23]. Another important aspect is that AHSCT is associated with quite a high mortality which seems likely the consequence of immunosuppression, usually more pronounced than that required for liver transplantation [27]. Recently, cirrhosis has been documented as a rare complication in a patient with MNGIE likely due to the accumulation of toxic intermediates in the liver [28]. Based on this case report, liver transplantation would be indicated not only as a source of TP, but also to prevent nucleoside-induced injuries [28].

The first quantification of TP activity has been obtained in animal models in the early 50′s by Friedkin and Roberts [25], [29]. Using the rabbit, they found that TP expression in the liver was less abundant than only that of the small intestinal mucosa, although greater than bone marrow > kidney > spleen. Heart, lung, skeletal muscle, and brain showed negligible activity [29]. Notably, the two authors demonstrated that liver TP activity was ∼6 times higher than those of the bone marrow (liver 10.0±2.9 vs. bone marrow 1.77±1.6 µM TP released h^−1^ g tissue^−1^) [29], the only tissue that has so far been proposed for transplantation in MNGIE patients. Friedkin and Roberts also demonstrated that TP is highly expressed in most mammalian (horse, cow, and rat) and non-mammalian (chicken) liver [25], [29]. Interestingly, in chicken embryos (5 to 18 days) most of the TP activity was found in the liver as compared to other tissues, suggesting that TP production during chicken embryogenesis is mainly dependent on the liver [29].

In this study, we have extensively characterized TP in human liver and demonstrated that TP is present in independent tissue protein extracts using both WB and ELISA. The WB approach provided evidence that TP is expressed in all analyzed samples, while the ELISA quantification revealed that TP content was 0.5 ng/μg total protein and varied significantly among subjects, confirming and expanding previous data by Yoshimura et al. [30]. Further immunohistochemical results demonstrated TP localization throughout the liver. Indeed, TP immunolabeling was detected not only (as expected) in the cytoplasm of hepatocytes, but also in the nuclei. This latter finding is interesting because it provides a morphological correlate for the well-known TP-mediated regulatory role in tissue proliferation and angiogenesis [31], [32]. TP activity was not measured in the investigated tissues as it is well known that TP protein expression correlates with its activity [33]. Furthermore, TYMP, formerly known as platelet-derived endothelial cell growth factor" (PD-ECGF), may exert a role in different types of tumors. In particular, TP expression has been found to be elevated in various solid tumors where it is likely involved in mechanisms that regulate cell proliferation, apoptosis, and angiogenesis [34], [35]. Finally, TP immunolabeling was also identified in sinusoidal lining Kupffer-like cells (i.e. possibly belonging to the mononuclear phagocyte system) [36]. However, the role exerted by TP in these specialized cells is still largely unknown.

Using WB we also compared TP levels in a number of normal tissues and found that the amount of TP measured in the duodenal mucosa was between that of liver and bone marrow. These findings are in line with finding of a dense infiltrate of TP immunopositive cells distributed mainly throughout the *lamina propria* and displaying features of immunocytes and fibroblasts. Normal buffy coats showed TP levels similar to the duodenal mucosa, but was not detectable in MNGIE buffy coats (used as negative control). Finally, TP immunoreactivity was undetectable in skeletal muscle, a finding that has previously been referred to as the ‘muscle paradox’ since this tissue is known to be a target of disease in MNGIE patients but does not contain TP [29], [30], [37], [38]. The explanation for the ‘muscle paradox’ remains unclear and this intriguing topic clearly requires further study.

Our data on TYMP transcript indicate endogenous synthesis and discard the possibility of TP uptake by the liver. TYMP mRNA is expressed in comparable amounts in bone marrow, liver and duodenal mucosa. No transcript was detected in skeletal muscle confirming the ‘muscle paradox’. Since TP protein concentrations vary in different tissues but is not associated with TYMP mRNA changes, it is conceivable that specific post-transcriptional regulation may occur.

In conclusion, our study demonstrates that the liver is a useful source of TP, six times higher than bone marrow. Thus, we propose orthotopic liver transplantation as a therapeutic alternative for MNGIE patients with a possible better outcome in terms of survival rate.
