# Correction: Liver as a Source for Thymidine Phosphorylase Replacement in Mitochondrial Neurogastrointestinal Encephalomyopathy

**DOI:** 10.1371/journal.pone.0110583

**Published:** 2014-10-06

**Authors:** The PLOS ONE Staff

There are errors in the author affiliations. The affiliations should appear as shown here:

Elisa Boschetti^1,2^, Roberto D’Alessandro^3^, Francesca Bianco^1^, Valerio Carelli^2,3^, Giovanna Cenacchi^2^, Antonio D. Pinna^1^, Massimo Del Gaudio^1^, Rita Rinaldi^4^, Vincenzo Stanghellini^1^, Loris Pironi^1^, Kerry Rhoden^1^, Vitaliano Tugnoli^2^, Carlo Casali^5^, and Roberto De Giorgio^1^

1 Department of Surgical and Medical Sciences, University of Bologna, Bologna, Italy, 2 Department of Biomedical and Neuromotor Sciences, University of Bologna, Bologna, Italy, 3 IRCCS Institute of Neurological Sciences, Bologna, Italy, 4 Neurology Unit, St. Orsola-Malpighi Hospital, Bologna, Italy, 5 Department of Medico-Surgical Sciences and Biotechnologies, University ‘La Sapienza’, Rome, Italy
